# Molecular and cytogenetic differentiation within the *Lariophagusdistinguendus* (Förster, 1841) species complex (Hymenoptera, Pteromalidae)

**DOI:** 10.3897/CompCytogen.v13i2.34492

**Published:** 2019-06-11

**Authors:** Christian König, Sina Paschke, Marie Pollmann, Ronja Reinisch, Cornelia Gantert, Justus Weber, Lars Krogmann, Johannes L.M. Steidle, Vladimir E. Gokhman

**Affiliations:** 1 Institute for Zoology, University of Hohenheim, Stuttgart, Germany University of Hohenheim Stuttgart Germany; 2 Department of Entomology, State Museum of Natural History Stuttgart, Germany State Museum of Natural History Stuttgart Germany; 3 Botanical Garden, Moscow State University, Moscow, Russia Moscow State University Moscow Russia

**Keywords:** Pteromalidae, *
Lariophagus distinguendus
*, cryptic species, phylogeny, COI sequencing, karyotype

## Abstract

Several strains of the apparently well-known cosmopolitan synanthropic parasitoid of coleopteran stored-product pests, *Lariophagusdistinguendus* (Förster, 1841) from Western Europe, were studied using DNA sequencing and chromosomal analysis. The presence of at least two cryptic species with different COI sequences and chromosome numbers (n = 5 and 6) was supported. The species with n = 6 is associated with the drugstore beetle *Stegobiumpaniceum* (Linnaeus, 1758), whereas the other one with n = 5 mostly develops on the granary weevil *Sitophilusgranarius* (Linnaeus, 1758). A phylogenetic study revealed that the karyotype with n = 6 represents an ancestral character state in this complex. Consequently, the chromosome set with n = 5 which is characteristic of a particular internal clade, apparently originated via chromosomal fusion which was probably preceded by a pericentric inversion. If this is true, inverted chromosome segments could accumulate a number of genetic loci responsible for certain interspecific differences.

## Introduction

Parasitoid Hymenoptera are among the most diverse, taxonomically complicated and economically important insect groups (Heraty 2017, Forbes et al. 2018). Over 80 thousand species of parasitoid wasps have already been described (Huber 2017). Furthermore, at least one million parasitoid species might still be unknown (Bebber et al. 2014, also see Quicke 1997). In addition to the poor knowledge of tropical fauna of parasitoid wasps, this high number of undescribed species apparently results from the phenomenon of the so-called cryptic lineages (Quicke 2002, Heraty 2017), which are very similar or virtually identical in morphology but differ considerably in genetic, ecological, behavioral, and other characteristics. Due to certain features of the parasitoid lifestyle, the latter phenomenon appears to be widespread among these insects (see Gokhman 2018 for review). Moreover, successful resolution of cryptic species complexes has important implications both for parasitoid wasp taxonomy and biological pest control (Heraty 2017).

The vast superfamily Chalcidoidea, which contains nearly 23 thousand described species (Huber 2017), is one of the largest groups among parasitoid Hymenoptera. Pteromalidae is one of the most species-rich chalcid families, comprising over 3,500 described species (Huber 2017). Although Pteromalidae*sensu lato* never recovers as a monophyletic group in all modern studies (see, e.g., Munro et al. 2011 and Heraty et al. 2013) and is going to be subdivided into a number of separate families, monophyly of the so-called pteromaloid complex, including Pteromalinae and few related subfamilies, has constantly been supported by recent cladistic analyses (e.g., Peters et al. 2018). Moreover, Pteromalinae include several known complexes of cryptic species. For example, the taxonomic revision of the genus *Anisopteromalus* Ruschka, 1912 has led to the description of a new cosmopolitan synanthropic species, *Anisopteromalusquinarius* Gokhman & Baur, 2014 which, together with the well-known *A.calandrae* (Howard, 1881) usually attacks various beetles that feed on stored products (Baur et al. 2014). Recently, cryptic lineages have also been detected in another cosmopolitan parasitoid from the subfamily Pteromalinae, *Lariophagusdistinguendus* (Förster, 1841) (König et al. 2015) with an analogous biology. Specifically, a particular lineage is apparently specialized on the drugstore beetle *Stegobiumpaniceum* (Linnaeus, 1758) (Coleoptera, Ptinidae) occurring in households, while strains of the other lineage were collected on weevils of the genus *Sitophilus* Schönherr, 1838 (Coleoptera, Dryophthoridae) in grain stores. To define the taxonomic status of these lineages, we have undertaken an extensive study of synanthropic populations of *L.distinguendus* from Western Europe using research of partial mitochondrial cytochrome oxidase I (COI) DNA sequences and chromosomal analysis. The results of this study are given below.

## Materials and methods

### Origin of parasitoid wasps

In total, fourteen strains of *L.distinguendus* were studied including nine strains described in König et al. (2015). Four new strains (CAN-D I, CAN-D III, OST-D I, and STU-D II) were collected by volunteers as part of a citizen science project in 2017 and 2018. In this project, bait boxes equipped with pellets of koi fish food (Hikari Friend, Kamihata Fish Industry Group, Kyorin Corporation, Japan) infested by *St.paniceum* were used. An additional strain (FRI-D I), also attacking *St.paniceum*, was sent to us by a private person. All strains were reared either on *St.paniceum* or *Sitophilusgranarius* (Linnaeus, 1758) depending on their host preferences, as described in König et al. (2015) (see Table 1 for the list of studied strains and specimens).

### DNA extraction and sequencing

DNA from *L.distinguendus* strains CAN-D I, CAN-D III, OST-D I, FRI-D I, BIR-D I and STU-D II was extracted and purified following the manufacturer’s instructions using Nexttec 1-Step DNA Isolation Kit – Tissue & Cell (Biozym, Hessisch Oldendorf, Germany) or DNeasy Blood & Tissue Kit (Qiagen, Hilden, Germany). PCR amplification, bidirectional sequencing, processing and editing of the partial COI fragment was performed as described in König et al. (2015). We used the primer pair C1-J-2183 5´-CAACATTTATTTTGATTTTTTGG-3´ and TL2-N-3014 5´-TCCAATGCACTAATCTGCCATATTA-3´ from Simon et al. (1994). The thermocycler program started with a denaturation temperature 95 °C / 2 min, followed by 40 cycles at 94 °C / 1 min, 58 °C / 1 min and 72 °C / 1.5 min. The final extension was 10 min at 72 °C. Positive PCR products were bidirectionally sequenced by Seqlab (Göttingen, Germany). Each chromatogram was checked for ambiguous positions and possible double peaks to avoid potential nuclear copies of mitochondrial sequences (NUMTs) (see Bensasson et al. 2001). All sequences were assembled using the program GENtle version 1.9.4 (by Magnus Manske, University of Cologne, Germany, released under GPL 2003). The obtained DNA sequences were translated into amino acid ones using the program “Virtual Ribosome” (Wernersson 2006) based on the code for invertebrate mitochondria to check for unexpected stop codons or gaps. The resulting consensus DNA sequences lacked ambiguity at all base pairs, and were finally aligned in MAFFT version 7 (Katoh and Standley 2013) with the G-INS-i algorithm (Katoh et al. 2005). Newly obtained sequences were submitted to GenBank (Table 1).

**Table 1.** Strains and specimens of *L.distinguendus* used in the molecular and chromosome study.

Strain	Host	Locality	Country/region	COI GenBank accession numbers	No. of specimens for chromosome study (male/female)	Haploid/diploid chromosome number
BIR-D I 1 BIR-D I 2	* St. paniceum *	Stuttgart Birkach	Germany/Baden-Württemberg	MK572719 MK572720	1(2) / 10(44)	6/12
BYG-DK I1 BYG-DK I2	* S. granarius *	Bygholm	Denmark	KJ867379 KJ867380	3(47) / 1(4)	5/10
CAN-D I1 CAN-D I2	* St. paniceum *	Stuttgart Bad Cannstatt	Germany/Baden-Württemberg	MK572723 MK572724	2(19) / 6(38)	6/12
CAN-D III 1	* St. paniceum *	Stuttgart Bad Cannstatt	Germany/Baden-Württemberg	MK572726	1(4) / 1(3)	6/12
FRI-D I1 FRI-D I2	* St. paniceum *	Fritzlar	Germany/Hessen	MK572717 MK572718	4(19+1^‡^) / 2(9)	6, 7^‡^/12
OST-D I2 OST-D I3	* St. paniceum *	Ostfildern	Germany/Baden-Württemberg	MK572721 MK572722	2(7) / 6(24+2^†^)	6/12, 13^†^
PFO-D I1 PFO-D I2	* S. granarius *	Pforzheim	Germany/Baden-Württemberg	KJ867383 KJ867384	4(32) / 2(10)	5/10
RAV-D I1 RAV-D I2	* St. paniceum *	Ravensburg	Germany/Baden-Württemberg	KJ867387 KJ867388	1(3) / 2(8)	6/12
SAC-D I1 SAC-D I2	* S. granarius *	Sachsen	Germany/Saxony	KJ867381 KJ867382	1(25) / 2(10)	5/10
SAT-D I1 SAT-D I2	* S. granarius *	Satrup	Germany/Schleswig-Holstein	KJ867375 KJ867376	1(10) / –	5/–
SLO-GB I1 SLO-GB I2	* S. granarius *	Slough	UK/Berkshire	KJ867377 KJ867378	4(28) / 1(13)	5/10
STU-D I1 STU-D I2	* St. paniceum *	Stuttgart Bad Cannstatt	Germany/Baden-Württemberg	KJ867385 KJ867386	2(18) / 1(1)	6/12
STU-D II1	* St. paniceum *	Stuttgart Mitte	Germany/Baden-Württemberg	MK572725	– / –	– / –
WAG-D I1 WAG-D I2	* St. paniceum *	Wageningen	The Netherlands	KJ867389 KJ867390	1(1) / 2(4)	6/12
–	–	F_1_ hybrids (RAV × PFO)	–		– / 6(29)	–/11
–	–	Male progeny of F_1_ hybrids	–		3+4(23+23) / –	5, 6/–

^†^An aberrant female karyotype with a smaller acrocentric fragment. ^‡^An aberrant male karyotype with an apparently fragmented acrocentric chromosome.

### Phylogenetic analysis

Phylogenetic analyses were conducted in MEGA X (Kumar et al. 2018) by first checking for the best-fit substitution model and subsequently constructing a maximum likelihood (ML) tree including 1000 bootstrap replications (Felsenstein 1985). Initial tree(s) for the heuristic search were obtained automatically by applying Neighbor-Join and BioNJ algorithms to a matrix of pairwise distances estimated using the Maximum Composite Likelihood (MCL) approach, and then selecting the topology with superior log likelihood value. The model for nucleotide substitutions [Hasegawa-Kishino-Yano (Hasegawa et al. 1985) allowing some sites to be evolutionarily invariable] was selected by applying the Bayesian Information Criterion (BIC) in MEGA X. The present analysis involved 27 nucleotide sequences and included 679 positions in the final dataset. Uncorrected *p*-distances were calculated using MEGA X.

### Chromosomal analysis

Chromosome preparations were obtained from cerebral ganglia of male and female prepupae of *L.distinguendus* following the protocol developed by Imai et al. (1988) with a few modifications (see e.g. Gokhman et al. 2017). Specifically, ganglia were extracted from insects dissected in 0.5% hypotonic sodium citrate solution containing 0.005% colchicine. The extracted ganglia were then transferred to a fresh portion of hypotonic solution and incubated for 30 min at room temperature. The material was transferred onto a pre-cleaned microscope slide using a Pasteur pipette and then gently flushed with Fixative I (glacial acetic acid: absolute ethanol: distilled water 3:3:4). The tissues were disrupted using dissecting needles in an additional drop of Fixative I. Another drop of Fixative II (glacial acetic acid: absolute ethanol 1:1) was applied to the center of the area, and the more aqueous phase was blotted off the edges of the slide. The slides were then dried for approximately half an hour and stored at room temperature. For chromosome staining, the preparations were usually left overnight in a freshly prepared 3% Giemsa solution in 0.05M Sorensen’s phosphate buffer (Na_2_HPO_4_ + KH_2_PO_4_, pH 6.8). Mitotic divisions were studied and photographed using an optic microscope Zeiss Axioskop 40 FL fitted with a digital camera AxioCam MRc (Carl Zeiss, Oberkochen, Germany). To obtain karyograms, the resulting images were prepared with image processing software: Zeiss AxioVision version 3.1 and Adobe Photoshop version 8.0. Mitotic chromosomes were measured on 20 haploid metaphases of each species using KaryoType software version 2.0. We report relative lengths (RL: 100 × length of each chromosome divided by total length of the set) and centromeric indices (CI: 100 × length of shorter arm divided by total length of a chromosome) for both species. On the karyograms, chromosomes were initially subdivided according to their measurements into elements characteristic of a particular chromosome set (columns 1–3) and those shared by the two main karyotypes (columns 4–7; see below). Within both groups, chromosomes were arranged in decreasing order of size. In addition, chromosomes were further classified into metacentric, subtelocentric or acrocentric according to the guidelines provided by Levan et al. (1964).

## Results

### Molecular data

**Figure 1.**
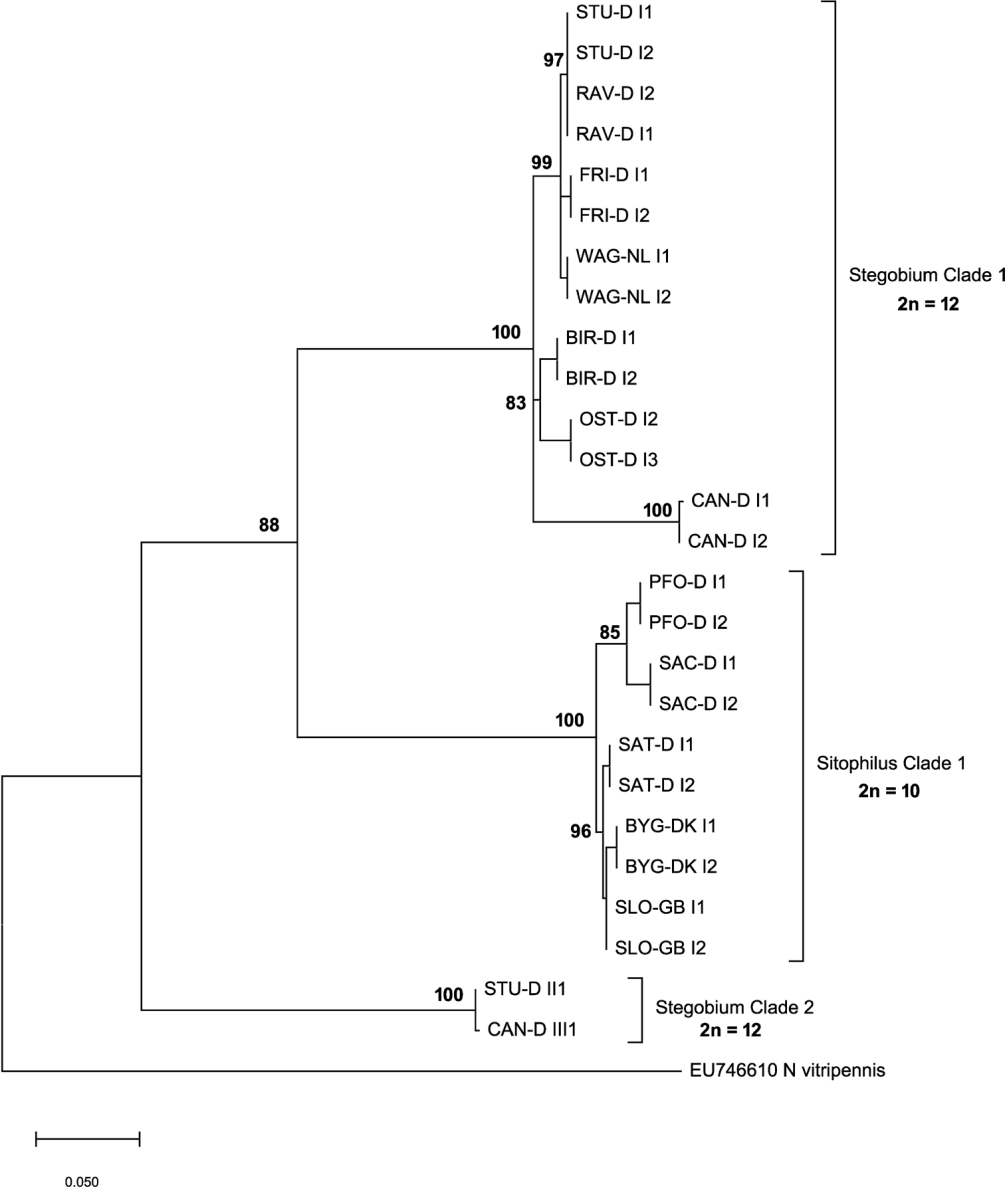
Evolutionary relationships of different strains of *L.distinguendus* based on a partial COI fragment. The evolutionary history was inferred by using the Maximum Likelihood method and Hasegawa-Kishino-Yano model (Hasegawa et al. 1985). The tree with the highest log likelihood (-2312.56) is shown. Percentages of replicate trees in which the associated taxa clustered together in the bootstrap test are shown next to the branches. The rate variation model allowed for some sites to be evolutionarily invariable ([+*I*], 65.23% sites). The tree is drawn to scale, with branch lengths measured in the number of substitutions per site.

Three main clades (*Stegobium* Clade 1, *Sitophilus* Clade 1, *Stegobium* Clade 2) were recovered within the *L.distinguendus* species complex (Fig. 1), including a particular one (*Stegobium* Clade 2) which can be considered as an outgroup to all previously studied strains (König et al. 2015). All strains from *Stegobium* Clades 1 and 2 were collected on *St.paniceum* in pantries or were trapped with *St.paniceum* samples as baits. In turn, all strains from the *Sitophilus* Clade 1 originate from samples from grain stores, which were infested with *S.granarius*. The average numbers of base differences per site for all sequence pairs of different clades were 0.137 between *Stegobium* Clade 1 and *Sitophilus* Clade 1, 0.155 between *Stegobium* Clades 1 and 2, and 0.147 between *Sitophilus* Clade 1 and *Stegobium* Clade 2. Sequence differences within the clades were low (*Stegobium* Clade 1 = 3.0%, *Sitophilus* Clade 1 = 1.6%, *Stegobium* Clade 2 = 0.1%).

### Cytogenetic data

**Figure 2.**
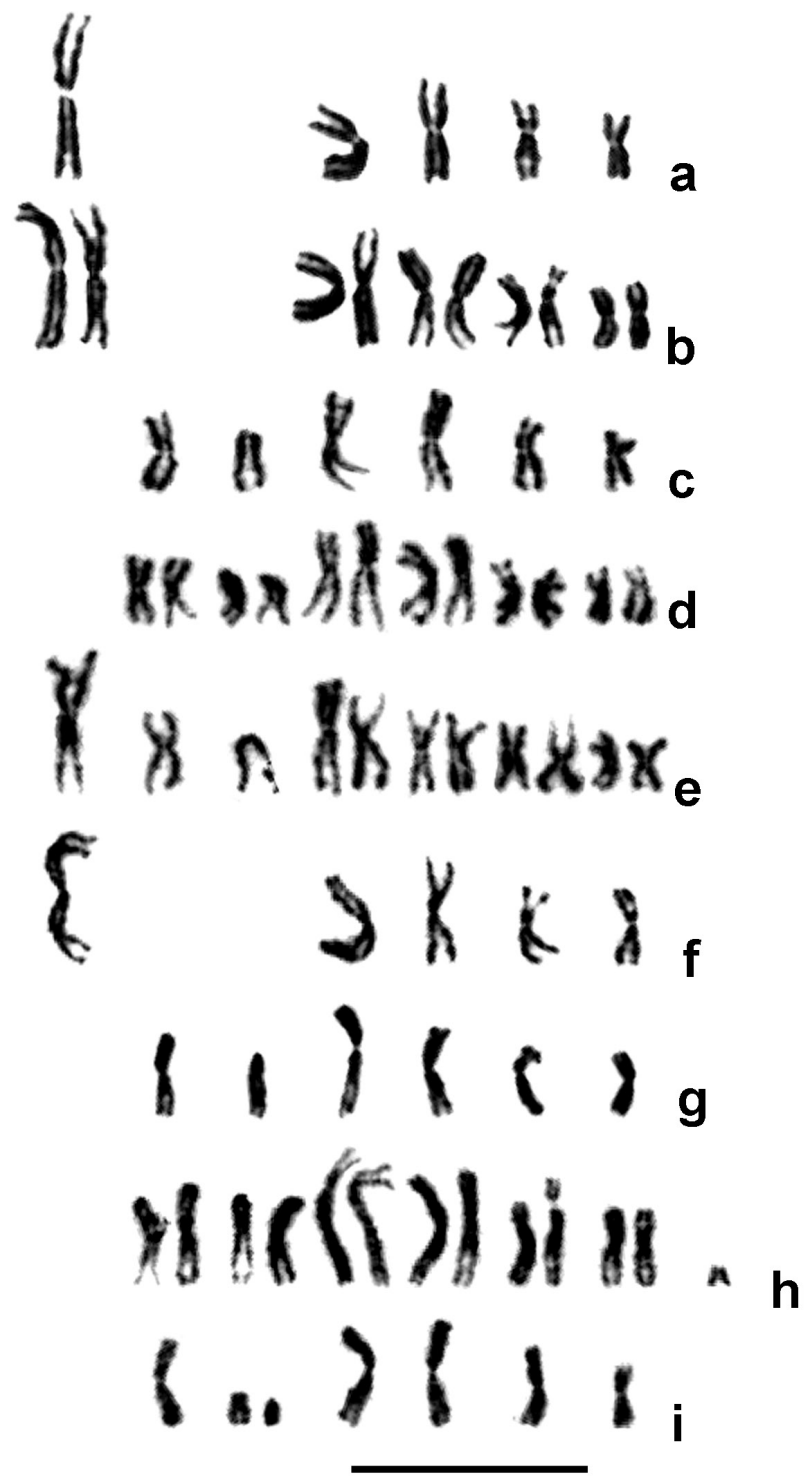
Karyotypes of different strains of the *Lariophagusdistinguendus* species complex (see Table 1 for details). **a** PFO-D I, male (n = 5) **b** SLO-GB I, female (2n = 10) **c** OST-D I, male (n = 6) **d** OST-D I, female (2n = 12) **e** F_1_ hybrid RAV-D I × PFO-DI, female (2n = 11) **f** progeny of F_1_ hybrid RAV-D I × PFO-D I, male (n = 5) **g** ditto (n = 6) **h** OST-D I, female, aberrant karyotype (the same individual as in **d** 2n = 13) **i** FRI-D I, male, aberrant karyotype (n = 7). Scale Bar: 10 μm.

**Figure 3.**
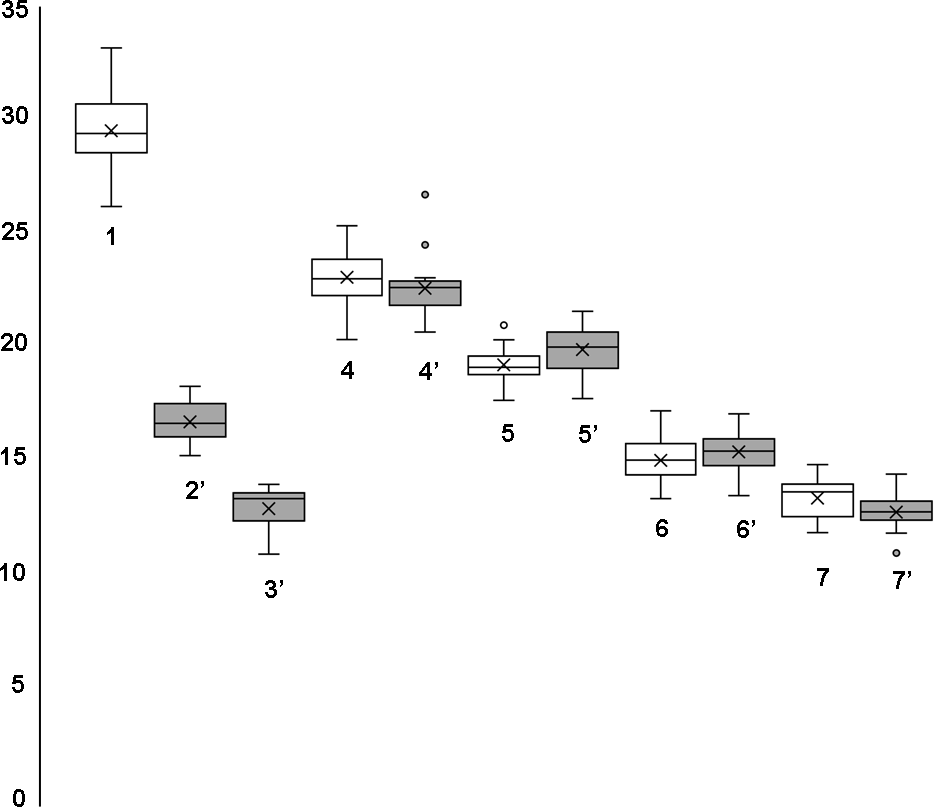
Box-and-whisker plot of relative lengths of chromosomes of different species of the *L.distinguendus* complex (based on data of the chromosome measurements also used in Table 2). The means, medians, second and third quartiles as well as variation ranges of RLs are represented by X signs, horizontal lines within boxes, boxes and whiskers respectively. 1, 4 etc. – numbers of chromosomes of the species with n = 5; 2’, 3’ etc. – numbers of chromosomes of the species with n = 6.

Chromosome study of all studied strains revealed two main karyotypes with different chromosome numbers, n = 5 (2n = 10) and 6 (2n = 12) (Fig. 2a–d). The karyotype of hybrid females contained 11 chromosomes (2n = 11), whereas their male progeny had either n = 5 or 6 (Fig. 2e–g). Preliminary measurements indicated that four metacentric chromosomes within both haploid karyotypes were similar. In addition, the karyotype with n = 5 contained the largest metacentric in the chromosome set, while the karyotype with n = 6 contained a smaller metacentric and the only acrocentric chromosome. These results were also confirmed by the detailed morphometric study (see Table 2 and Fig. 3). Moreover, four similar metacentrics were clearly paired within female karyotypes of F_1_ hybrids, whereas the other three elements were represented by single copies (Fig. 2e). This suggests that certain unpaired chromosomes from different karyotypes correspond to each other. This assumption is further corroborated by the fact that combined RLs of the two smaller chromosomes (no. 2 and 3) in the karyotype with n = 6 were almost exactly equal to the RL of the largest metacentric (chromosome 1) in the karyotype with n = 5 (see Table 2). In addition, these chromosomes were again segregated in the male progeny of F_1_ hybrid females that contained both karyotypes with n = 5 and 6 in similar proportions (Table 1, Fig. 2f–g).

A few aberrant mitotic divisions were also detected. Specifically, most metaphase plates from a particular female individual of OST-D I strain had the normal karyotype with 2n = 12 (Fig. 2d), whereas a few cells carried a small additional, apparently acrocentric element (Fig. 2h). On the other hand, almost all metaphase plates of another male specimen of FRI-D I strain also showed a normal chromosome set with n = 6, although a single mitotic division with n = 7 was found (Fig. 2i). A detailed study of the latter karyotype suggests that it carries two smaller elements, a subtelocentric and an acrocentric. In this case, chromosome morphometrics demonstrates that the two chromosomes probably result from fragmentation of the medium-sized acrocentric of the normal karyotype.

**Table 2.** Measurements of mitotic chromosomes on haploid metaphase plates of the *L.distinguendus* complex with n = 5 and 6 (N = 20; mean ± SD).

Karyotype / chromosome no.		1	2	3	4	5	6	7
n = 5	RL	29.48 ± 1.77	–	–	23.03 ± 1.15	19.19 ± 0.75	14.98 ± 0.91	13.32 ± 0.86
	CI	47.06 ± 3.16	–	–	46.13 ± 2.25	47.25 ± 1.79	43.74 ± 3.83	44.49 ± 4.58
n = 6	RL	–	16.68 ± 0.89	12.86 ± 0.94	22.55 ± 1.28	19.86 ± 1.05	15.35 ± 0.83	12.70 ± 0.83
	CI	–	45.45 ± 4.08	0	43.27 ± 3.30	46.96 ± 2.69	45.83 ± 2.64	46.10 ± 3.23

## Discussion

### Molecular phylogeny

Phylogenetic analysis of COI sequences revealed a clear separation of the strains into three main clades, supported by high bootstrap values (Fig. 1). The molecular divergence between the clades was remarkably high (13.7% – 15.5%) in contrast to the low genetic differences within the clades. Interestingly, the position of the strains in the cladogram was correlated with their host preference, and was independent of their geographic origin (Table 1). All strains from *Stegobium* Clades 1 and 2 were associated with drugstore beetles (*St.paniceum*), whereas all strains from *Sitophilus* Clade 1 were collected on weevils of the genus *Sitophilus* in grain stores (König et al. 2015). The fact that *Stegobium* Clade 2 is basal to all other main clades suggests that *St.paniceum* or a closely related species can be the ancestral host, and that *Sitophilus* Clade 1 evolved by a host shift to *Sitophilus*. This agrees with the hypothesis by König et al. (2015) on the evolution of the two cryptic lineages of *L.distinguendus*. Remarkably, this host shift was probably related to the ability to learn host-related cues (König et al. 2015).

### Chromosome study

Apart from a few aberrant metaphase plates, two main karyotypes with n = 5 (2n = 10) and n = 6 (2n = 12) were detected. Specifically, the latter chromosome set is characteristic of the strains of *Stegobium* Clades 1 and 2 which originated from samples developing on *St.paniceum*, while karyotype with n = 5 was found in all members of *Sitophilus* Clade 1 from grain stores which were associated with weevils of the genus *Sitophilus* (König et al. 2015; Fig. 1, also see above). These data indicate that n = 6 is the ancestral character state for the *L.distinguendus* species complex, and the chromosome set with n = 5 is derived, although this is in contrast to the idea that n = 5 is apparently ancestral for members of Pteromalidae (Gokhman 2009). Chromosome measurements (Table 2) indicate that the karyotype with n = 5 in *L.distinguendus* most likely originated from fusion of chromosomes 2 and 3 of the karyotype with n = 6, yielding chromosome 1, the largest metacentric in the karyotype with n = 5. Together with some other recent studies (see e.g. Gokhman et al. 2017), the present work thus demonstrates substantial importance of molecular research for the phylogenetic reconstruction of karyotype evolution of parasitoid wasps.

Our recent hypothesis that the decrease in the chromosome number in the *L.distinguendus* species complex occurred through chromosomal fusion is further corroborated by the results of the karyotypic study of F_1_ hybrids between these forms (Fig. 2e). As far as possible rearrangements underlying the above-mentioned decrease in the chromosome number are concerned, either central or tandem chromosomal fusion can be proposed (White 1973, Gokhman 2009). In the case of central fusion, it must be preceded by a pericentric inversion in the smaller metacentric of the chromosome set with n = 6. If this is true, the two species of the *L.distinguendus* complex also differ by this inversion, in addition to the chromosomal fusion. Interestingly, accumulation of genetic loci responsible for certain differences between closely related forms within inverted chromosomal segments now became a key feature of the so-called “supergene concept”, a popular approach in modern evolutionary genetics (see e.g. Thompson and Jiggins 2014). This concept is based on the fact that chromosome inversions interfere with the process of crossing-over, thus preventing recombination within the inverted segments (White 1973). Nevertheless, one-step rearrangement, i.e., a tandem fusion between the acrocentric and the metacentric chromosome accompanied by centromere inactivation in the longer arm of the resulting larger metacentric, is also possible (White 1973, Gokhman 2009).

### Taxonomic implications of the molecular and cytogenetic studies

All obtained information, together with data on reproductive relationships and host specificity of the studied strains (König et al. 2015), suggests that the *L.distinguendus* complex harbors at least two cryptic species. However, no reliable morphological difference between these species was found to date (Wendt et al. 2014). This information, as well as their karyotypic similarity and the possibility of interspecific hybridization indicates that these cryptic species are closer to each other than e.g. those of the genus *Anisopteromalus* (Baur et al. 2014). Nevertheless, genetic differences between members of the *L.distinguendus* complex together with our preliminary data on the decreased production of hybrid offspring from crossings between forms with different karyotypes confirm that this complex harbors separate species. Our results thus describe the first case of hybridization between two cryptic parasitoid species with different chromosome numbers. On the other hand, relatively strong differences in the structure of COI sequences between certain strains with the same karyotype do not necessarily indicate their species status (see e.g. Hernández-López et al. 2012, Korenko et al. 2018). Further molecular studies, which should also include nuclear markers for those strains that were not previously examined in this respect, are therefore needed (see König et al. 2015).
